# Constructing Compact Signatures for Individual Fingerprinting of Brain Connectomes

**DOI:** 10.3389/fnins.2021.549322

**Published:** 2021-04-06

**Authors:** Vikram Ravindra, Petros Drineas, Ananth Grama

**Affiliations:** Department of Computer Science, Purdue University, West Lafayette, IN, United States

**Keywords:** fingerprinting, functional connectomics, matrix sampling, dimensionality reduction, randomized numerical linear algebra

## Abstract

Recent neuroimaging studies have shown that functional connectomes are unique to individuals, i.e., two distinct fMRIs taken over different sessions of the same subject are more similar in terms of their connectomes than those from two different subjects. In this study, we present new results that identify specific parts of resting state and task-specific connectomes that are responsible for the unique signatures. We show that a very small part of the connectome can be used to derive features for discriminating between individuals. A network of these features is shown to achieve excellent training and test accuracy in matching imaging datasets. We show that these features are statistically significant, robust to perturbations, invariant across populations, and are localized to a small number of structural regions of the brain. Furthermore, we show that for task-specific connectomes, the regions identified by our method are consistent with their known functional characterization. We present a new matrix sampling technique to derive computationally efficient and accurate methods for identifying the discriminating sub-connectome and support all of our claims using state-of-the-art statistical tests and computational techniques.

## 1. Introduction

A number of functional MRI studies focus on differences between sets of cohorts (e.g., healthy vs. diseased). These studies characterize an invariant signal within each cohort and identify significant changes in these signals across cohorts. Invariant signals within cohorts are designed to suppress individual-level heterogeneity of the functional connectomes, and can be computed using methods that range from simple averaging to sophisticated matching techniques, to identify statistically significantly conserved components within the cohort. However, it has long been known that structural and functional differences are observable within a cohort of patients (Rypma and D'Esposito, 1999; Amunts et al., 2000; Newman et al., 2003; Mangin et al., 2004). Moreover, a number of studies suggest that individual differences among healthy subjects reveal interesting, potentially important patterns. For instance, IQ scores are shown to be positively correlated with smaller path lengths (Li et al., 2009), and with higher degree of connectivity in the prefrontal cortex (Cole et al., 2012).

Clinically observed functional and/or structural variability between individual brains typically manifests in structural and functional connectomes. It has been shown that functional connectomes of an individual, taken over multiple sessions, express higher degree of similarity than connectomes of different individuals (Mueller et al., 2013; Miranda-Dominguez et al., 2014; Finn et al., 2015; Amico and Goñi, 2018; Byrge and Kennedy, 2019).

It has also been shown that restricting such analyses to regions constituting well-known networks, such as fronto-parietal networks, amplifies the individual-specificity (Finn et al., 2015). This suggests that the signal corresponding to identifiability is spatially selective in its expression. We refer to this individual-specific signal as a *signature*. Such signatures are persistent underlying signals that sustain over a period of several days, while being robust to temporally local fluctuations in brain activity.

In this paper, we present a technique for automatically identifying *interpretable* individual-specific signatures in resting and functional brain networks. Our method finds a parsimonious representation of functional connectomes by identifying a small subset of features (which are subsequently mapped to localized regions on the cortex). We show that this compact representation accurately captures uniqueness of individuals, as reflected in high accuracy of identification. Furthermore, we demonstrate the statistical significance and robustness of our signatures, by showing that the signatures correspond to regions of the brain that are consistent across subjects. The strengths of our feature selection method are: (i) Our method does not require any prior knowledge of the functional aspects of the brain other than parcellation; (ii) We achieve significant feature-set reduction by selecting a small fraction of edges that represents the entire connectome; (iii) Our method has well-characterized performance guarantees, and can therefore potentially serve as a generic feature-selection strategy for finding neuromarkers in other cohort-level studies; and (iv) Our feature selection method requires only one session of data, which makes it useful for datasets without test-retest sessions.

## 2. Methods and Materials

### 2.1. Dataset

The images used in this study are collected as part of the Healthy Young Adult study in the WU-Minn Project (Essen et al., 2013) by the Human Connectome Project Consortium. The data includes 3T structural and functional MRI for 1,113 adults, 7T resting and task magnetoencephalography (MEG) from 184 subjects, and 3T and 7T diffusion data. Full details of the data can be found in Essen et al. (2012).

The resting state functional MRI is acquired in two sessions, on two separate days. The resting state data in each session lasted ~30 min [15 min for left right (L-R) and right left (R-L) phase encoding]. The directions are those of the of the magnetic gradient. Further details on selecting parameters for MRI scanners can be found in the HCP reference manual (WU-Minn, 2017). The spatial resolution was 2 × 2 × 2 *mm*^3^ and temporal resolution (TR) of 720 ms. For a detailed description of the acquisition protocol see Smith et al. (2013). In each session, the resting state session was followed by tasks. On the first day, the tasks included working memory, gambling, and motor; the second day included language, social cognition, relational processing, and emotional processing. The task functional MRI data also have the L-R encoding and the R-L encoding. However, the duration of each session varied considerably, ranging from 176 frames in emotion processing to 401 frames in working memory, with TR of 5,520 ms. For a detailed description of the protocol see Barch et al. (2013).

#### 2.1.1. Pre-processing

The preprocessing steps follow the HCP minimum pre-processing pipeline prescribed by Glasser et al. (2013) and Smith et al. (2013). The HCP pre-processing steps include spatial pre-processing and temporal pre-processing. In resting state functional MRIs, we also perform global signal regression.

The pipeline includes procedures for removal of spatial artifacts and distortion, head motion correction, and co-registration to structural image and normalization to the standard space. We perform spatial preprocessing, limiting spatial smoothing only to the surface vertices, as described in Smith et al. (2013). We perform temporal preprocessing with a weak highpass filter (>2,000s full width at half maximum), which removes slow drift. The artifact due to subject motion was regressed out using 6 DOF (using FSL FLIRT). The final registration to CIFTI coordinates is performed by the fMRISurface pipeline. The quality control procedure for the images is explained in Marcus et al. (2013). We do not deviate from the standard pipeline^1^, so the data we use can be recreated by running the three structural pipelines—Pre-FreeSurfer, FreeSurfer, and Post-FreeSurfer and the two functional pipelines—fMRIVolume and fMRISurface.

In the case of resting state functional MRI, we follow this by global signal regression, a procedure that regresses out the mean time series out of every time-series. Since resting state analyses have shown that fluctuations at low frequencies are due to hemodynamic responses to neural activation, we apply a bandpass filter (0.008–0.1 Hz). We do not apply the bandpass filter to task fMRIs since it is unclear what the best frequency ranges are for different tasks, as described in Cole et al. (2014).

#### 2.1.2. Brain Atlas

In our work, the cortical structures are parcellated in accordance with the atlas of Glasser et al. (2016). This atlas consists of 360 regions (180 in each hemisphere), bound by sharp edges on the basis of anatomy, function, and/or topology. Each of the 180 regions are also classified into 22 larger regions in Glasser et al. (2016), which allows for analysis at a coarser level, if needed. We find that other parcellation schemes either have too few regions, or are annotated on the basis of fewer samples. In contrast, the boundaries in Glasser et al. (2016) are drawn using resting state and task fMRI images from two groups of 210 subjects, all of which is part of the previously described Human Connectome Project.

### 2.2. Methodology

#### 2.2.1. Experimental Setup

The time series data is z-score normalized. Following this, it is parcellated to yield a *region* × *time-points* matrix. The Pearson Correlation of all pairs of time series is then computed, resulting in a *region* × *region* correlation matrix. This process is repeated for both *groups* (defined below) of all subjects. Then, the upper triangular matrix for the first group for all subjects is vectorized and stacked next to each other into a *subjects* × *feature* matrix, where a feature corresponds to an entry in the correlation matrix. A similar matrix is constructed for the second group as well.

In the first set of experiments, where we use resting state connectomes, the two groups have correlation matrices from REST1 and REST2 sessions, respectively. In the experiment that investigates identifiability of individuals while performing different tasks, the first group consists of the first half of the session (RL encoding) and the second group consists of the last half of the session (LR encoding). In the final experiment that focuses on task identifiability, the first group consists of REST1 and the RL encodings of seven tasks, whereas the second group consists of REST2 and the LR encodings of the same seven tasks. Each group matrix is constructed by stacking the vectorized correlation matrices. Thus, each data point in this matrix corresponds to a subject and each feature is a measure of coherence in activation between two regions. For simplicity, we refer to the group matrices as *G*_1_ and *G*_2_ in every experiment. Our task is to successfully match the functional connectomes (i.e., the *regions* × *regions* Pearson Correlation matrices) belonging to a subject across the two groups.

We first discuss the concept of row and column sampling as a general means of feature selection and, in particular, leverage score sampling. We then discuss the use of leverage scores to select features for brain fingerprinting.

#### 2.2.2. Row/Column Sampling

Given matrix *A*, an individual entry *a*_*i, j*_ corresponds to the weight of the *i*th edge of subject *j*. The problem of identifying the most discriminating subset of features then translates to selecting non-zeros from the correlation matrix (row-column pairs) that are most descriptive in terms of individual signatures. In contrast to conventional dimension reduction techniques, such as Singular Value Decomposition (SVD), the ability to choose rows or columns from a data matrix directly translates to *feature selection*, especially when features have physical meaning. Retaining such features in the matrix sketch can make the underlying physical phenomenon explainable, while also de-noising the data by eliminating non-discriminating features and noise. Furthermore, in contrast to methods that use within-subject and between-subject metrics to reduce dimensions as in Byrge and Kennedy (2019), we do not require data from both sessions to identify important edges.

Given a matrix *A* ∈ ℝ^*m* × *n*^, one can use the following randomized meta algorithm to create a sketch matrix Ã that retains *s* (< *m*) rows.

**Table:** 

**function** row_sample(A,s)
Let *Ã* be an empty matrix
**for** *t* = 1 to *s* **do**
Randomly sample a row according to the distribution *P*
Let *A*_*i*_*t*_, ⋆_ be the sampled row, with corresponding probability *p*_*i*_
Set ${\tilde{A}}_{t,⋆}=\frac{1}{\sqrt{sp_{i}}}A_{i_{t},⋆}$
**end for**
**return** *Ã* and row indices
**end function**

The algorithm samples *s* rows of *A* in independent, identically distributed trials according to *P*s. The re-scaling of Ã ensures that Ã^*T*^Ã is unbiased, i.e., $𝔼\left[{\left({\tilde{A}}^{T}A\right)}_{i,j}\right]={\left(A^{T}A\right)}_{i,j},\forall i\in \left\{1\ldots m\right\},j\in \left\{1\ldots n\right\}$ (Drineas et al., 2006).

The key unspecified detail is the choice of distribution *P*. A simple choice is to sample rows uniformly, however, this yields poor results. An intuitive choice for the distribution relies on the matrix *A* itself—assigning higher weights to more important elements. A non-uniform distribution is based on *l*_2_ sampling, which can be defined as:

(1) $$\begin{array}{r} p_{i}=\frac{||A_{i,⋆}{||}_{2}^{2}}{\sum_{i}||A_{i,⋆}{||}_{2}^{2}}=\frac{||A_{i,⋆}{||}_{2}^{2}}{||A_{i,⋆}{||}_{F}}. \end{array}$$

Using norm-squared sampling, Drineas et al. (2006) prove that:

(2) $$\begin{array}{r} 𝔼\left[||A^{T}A-{\tilde{A}}^{T}\tilde{A}{||}_{F}\right]\leq \frac{1}{\sqrt{s}}||A{||}_{F}^{2}. \end{array}$$

The bounds in Equation (2) imply that Ã can be used as a proxy for *A*. However, this approximation introduces an additive error, which depends on ||*A*||_*F*_. To achieve better bounds, one can make use of the knowledge of column space of *A*. The associated sampling technique is called leverage score sampling (Drineas and Mahoney, 2016).

### 2.3. Leverage Score Sampling

Let *A* ∈ ℝ^*m* × *n*^, with *m* ≫ *n*. Let *U* ∈ ℝ^*m* × *n*^ be the left singular matrix spanning the column space of *A*. Then, *U*^*T*^*U* = *I* and $UU^{T}=P_{A}$, which is an *m*-dimensional projection matrix onto the span of *A*. Then, the probabilities *p*_*i*_ are defined as:

(3) $$\begin{array}{r} p_{i}=\frac{||U_{i,⋆}{||}_{2}^{2}}{\sum_{i}||U_{i,⋆}{||}_{2}^{2}}=\frac{1}{n}{\left(P_{A}\right)}_{i,i}\;\forall i\in \left\{1\ldots m\right\}. \end{array}$$

The values of *p*_*i*_s in Equation 3 are known as *statistical leverage scores*. If we select $O\left(k\log k/\epsilon^{2}\right)$ rows, we get a relative error bound as follows:

(4) $$\begin{array}{r} ||A-A{\tilde{A}}^{†}\tilde{A}{||}_{\zeta }^{2}\leq \left(1+\epsilon \right)||A-A_{k}{||}_{\zeta }^{2}, \end{array}$$

where ζ ∈ {2, *F*}, ϵ ∈ [0, 1/2), *A*_*k*_ is the best rank-k approximation and † represents the pseudo-inverse (Drineas et al., 2008).

In our matrix of vectorized edge values, each row contains values expressing functional connectivity markers between the same pair of physical regions on the brain cortex. Hence, leverage scores are indicative of relative importance of edges in discriminating samples. While the randomized approach discussed here helps in understanding the process, we find that a deterministic approach performs well in practice. We call this sketching process *Principal Features Subspace Method*.

#### 2.3.1. Principal Features Subspace Method

As before, let *A* be the data matrix of connectomes, and *U* be the orthonormal matrix that spans the column space of *A*. Additionally, let *t* be the number of features that need to be retained. An example of such a matrix *U* is constructed using left singular vectors from a Singular Value Decomposition (SVD) of *A*. We can then compute the leverage(*l*) scores of *A* as:

(5) $$\begin{array}{r} l_{i}=||U_{i,⋆}{||}_{2}^{2},\;\forall i\in \left\{1\ldots m\right\}. \end{array}$$

We sort the leverage scores and retain the features corresponding to the top *t* leverage scores. We call this subspace the *principal features subspace*. In contrast to prior randomized approaches, we select features in a deterministic manner; Cohen et al. (2015) provide theoretical bounds for this selection process. The main advantage of using leverage scores is that the features representing high leverage (in the left-singular vector space) correspond to highly representative (in other words, important) features in the data space. We do not project the vectors in the linear algebraic sense, but rather, we restrict the feature space by selecting a small subset of highly expressive features. To this end, our de-identification pipeline is not directly based on SVD, rather it is based on use of leverage score sampling.

#### 2.3.2. Our Approach

Starting from the matrix of vectorized correlation values *G*_1_, we compute the left singular vectors using SVD. We compute the leverage scores of the rows of *G*_1_ and retain features with high leverage scores. We show that the task of predicting matching columns of *G*_1_ and *G*_2_ can be efficiently achieved by restricting to the small set of retained features. The ordering of edges according to their leverage scores, if robust across different groups, is indicative of a set of features that can accurately fingerprint an individual's functional connectome. In this case, for a given parcellation scheme, and for a given measure of region-region coherence, we need to apply SVD just once to determine the relevant edges. We present results from our scheme applied to 100 fMRI samples to demonstrate powerful new results on the compactness and robustness of the sub-connectome coding individual fingerprints.

### 2.4. Related Methods

There is significant recent work in characterizing population-level differences from neuroimaging datasets. Given the focus of our work on individual-level differences, we restrict ourselves to significant results in this space, and use them to motivate our new results.

A simple linear model was proposed by Miranda-Dominguez et al. (2014), wherein the activity of a given brain region are described by the weighted sum of its functional neighboring regions. This individual-specific, model-based connectivity matrix was shown to be capable of predicting the time-series of each subject at a later time. Finn et al. (2015) divide the identification task into two steps: whole brain and network specific. In the first step, they perform identification on the basis of the region-wise correlation matrix of the whole brain, and report a success rate of ~93%. Following this, they divide the brain into eight functional networks: medial frontal, fronto-parietal, default mode, subcortical-cerebellum, motor, visual I, visual II, and visual association, on the basis of an atlas constructed from the Yale Dataset (268 nodes covering the whole brain). Restricting analysis to each of these regions, they perform the identification task. From this analysis, they find that the networks medial frontal and fronto parietal carry most discriminating features, yielding accuracies as high as 98%. Similar results have also been reported by Mueller et al. (2013) and Miranda-Dominguez et al. (2014). However, these results require significant prior knowledge on composite of parcels known to have functional/ structural coherence. The stability of individual differences over time and the independence from task associated states was studied by Gratton et al. (2018) using high-quality, highly-sampled, and long-acquisition-time dataset created by Gordon et al. (2017). They conclude that functional networks are indeed suitable for measuring individual differences, and can therefore be used in precision psychiatry or personalized medicine.

Our effort takes a similar two-level approach, viewing identification at the whole brain and network levels. Instead of using prior knowledge, we show that the *Principal Features Subspace* yields a comparable identification training accuracy of ~96%. Using the features obtained in the training set on the test set, we find that our test accuracy is around 93%, which is significantly higher than that from Finn et al. (2015). In contrast to Finn et al., we restrict the analysis in the second step to the regions that are computationally identified from our first step. The regions obtained from our first step are in fact largely consistent with those used by Finn et al. in their second step, in that we find regions in the parietal and frontal cortex being over-represented in our top nodes (nine out of 12 belong to fronto-parietal regions). However, the network identified by our method is significantly smaller than the region-specific network of Finn et al., and is shown to be robust, statistically significant, and invariant on test subjects. Restricting ourselves to 24 parcels (which may belong to different networks), we report an accuracy of ~96%.

There have been recent attempts aimed at identifying discriminating edges, using the so-called *differential power* introduced by Finn et al. and improved on by Byrge and Kennedy (2019). Finn et al. define differential power as follows: given the set of connectomes (C) of all sessions ($S\in \left\{1,\ldots ,n_{sessions}\right\}$) of all subjects ($P\in \left\{1,\ldots ,n_{subjects}\right\}$), the following quantity is calculated for each edge *e*, and for all pairs of subjects $\phi_{i,j}(e)=vec(C_{i,j}^{k})(e))\times vec(C_{i,j}^{k^{\prime }})(e),\forall i,j\in P;k,k^{\prime }\in S;C\in C.$ Here, *vec*() vectorizes the matrix **C**. An edge is considered to be expressive if ϕ_*ii*_ > ϕ_*ij*_, or ϕ_*ii*_ > ϕ*ji*, i.e., similarity across sessions for a subject is higher than similarity across subjects. Then, the probability of an edge *e* being important for a subject *i* is defined as $P{\left(e\right)}_{i}\propto \underset{i}{\sum }\frac{𝟙\left[\phi_{ii}>\phi_{ij}\right]+𝟙\left[\phi_{ii}>\phi_{ji}\right]}{O\left(n_{subjects}^{2}\right)},\forall i,j\in P$. Finally, the differential power of an edge over a population is given by: $DP\left(e\right)=\underset{i}{\sum }-\ln P_{i}\left(e\right)$. Building on this definition, Byrge et al. define differential power as follows: first, they compute across-subject variability as the mean (across sessions) of the standard deviation of the functional connectome value for every feature across subjects within a given session, and within-subject variability as the mean (across subjects) of the standard deviation of the functional connectome value for every feature across sessions within each subject. The features with highest variability ratio (ratio of across- and within-subject variability) are considered most expressive. Byrge et al. compute variability in terms of both first and second order moments, which work well in practice. The main difference in the aims of Finn et al. and Byrge et al. is that while the former used two sessions of resting-state and task-based images to correctly identify pairs of connectomes belonging to the same subject, the latter is test-retest study that focuses on resting data. In comparison to Byrge et al., our approach has two distinct advantages. First, our *principal features subspace* method uses the concept of leverage scores, which has strong theoretical guarantees (Drineas et al., 2006; Papailiopoulos et al., 2014; Cohen et al., 2015; Drineas and Mahoney, 2016). Second, our approach does not require any labels in order to find discriminating edges. This significantly generalizes the applicability of our method, since most studies do not have multiple scans of a subject. Since acquisition protocols may vary from study to study, it is not always possible to use the features from HCP in other studies, where it may be necessary to find subject-level discriminating patterns. Our setup simply requires two sets of images, with the guarantee that each subject has one scan in each set. We use only one set to find discriminative edges and the second set to establish the efficacy of our approach. In this context, the applicability of differential power is unclear.

Column/ row sampling using randomized techniques has significant benefits, in addition to de-noising and avoiding overfitting. Other commonly used de-noising techniques transform the data matrix into a combination of linearly independent (orthogonal) components, using methods, such as Principal Component Analysis (PCA) or Singular Value Decomposition (SVD). De-noising can be achieved by discarding the components corresponding to noise, and working with a low rank approximation of the matrix, as explored in context of brain fingerprinting by Amico and Goñi (2018).

Using the “unrelated” subset of HCP subjects, Amico and Goñi (2018) report that using the original data matrix (simply computing correlations with known samples and selecting the most correlated subject for identification) as a fingerprint yielded an accuracy of around 87%. The process of de-noising using low rank approximations yields better results—Amico and Goñi (2018) report an accuracy of 91% for brain fingerprinting resting state fMRIs. Along the lines of Amico and Goñi (2018), Ferguson et al. (2017) also explore the relationship between spectral decompositions and identifiability. They find the correlation between the top singular vector space and various task metrics. Moreover, they find spatial patterns of functional synchrony across brain regions in rank-1 approximations of connectomes built by the spectral decompositions of the *regions* × *regions* correlation matrices.

In a related work, Airan et al. (2016) discuss various factors related to acquisition protocols that affect identifiability, such as length of acquisition, sampling frequency, time course extraction, region of interest parcellation, and thresholding of connectivity-derived network graphs. They report that there is a tradeoff between sampling frequency and image acquisition time, and that 3–4 min worth of images suffice to accurately identify individuals.

As discussed earlier, the advantage of the *principal features subspace* method is that it provides a small set of regions, thereby de-noising data, while also making the connectivity matrix much smaller. It also identifies the signature as a set of biologically interpretable features.

## 3. Results

We characterize the uniqueness of the functional connectome of an individual in terms of its accuracy, underlying features, and associated task-specificity. We define the innate uniqueness property as identifiability. We define a *signature* as a pattern that encodes identifiability. In this effort, we isolate small regions in the human cortex that strongly express these signatures. In this context, we use the terms *signatures* and *fingerprints* interchangeably. We present detailed experiments to characterize the accuracy of these signatures in terms of their ability to identify individuals, as well as the task they are performing.

We used the functional MRI images of the unrelated subset of subjects (54 females, 46 males, mean age = 29.1 ± 3.7 years), acquired as part of the WU-Minn Project by the Human Connectome Project (HCP) Consortium. Please refer to the Online Methods for more details regarding the dataset and preprocessing steps. In each our experiments, we use two groups (*G*_1_ and *G*_2_) of connectomes (please see Methods). Each group has exactly one data point per subject. Our task is to correctly identify pairs of columns of *G*_1_ and *G*_2_ belonging to the same subject. We define *accuracy of identification* as the percentage of instances where individuals are correctly matched across the two groups, *G*_1_ and *G*_2_. Note that a match is identified as the highest correlation of an individual in *G*_2_ over all individuals in *G*_1_.

### 3.1. A Small Set of Features Encode Individual Signatures

Our first set of experiments is designed to demonstrate our first result, that a small number of features encode resting-state signatures that are largely unique to individuals. This is in contrast to prior techniques that do not identify either structural or functional features associated with individual signatures; rather they demonstrate the existence of such signatures in the aggregate datasets.

To demonstrate this result, we use the group matrix *G*_1_ to identify features corresponding to the top 100 leverage scores (from among over 64K scores). We show later that accuracy of identification saturates at <100 features. Then, pairwise correlation scores are calculated between data points of the two groups in this restricted feature-space. The purpose of this experiment is to demonstrate that two Functional Connectomes of the same subject are more similar than two Functional Connectomes belonging to different subjects using only this small feature set.

**Figure 1:**
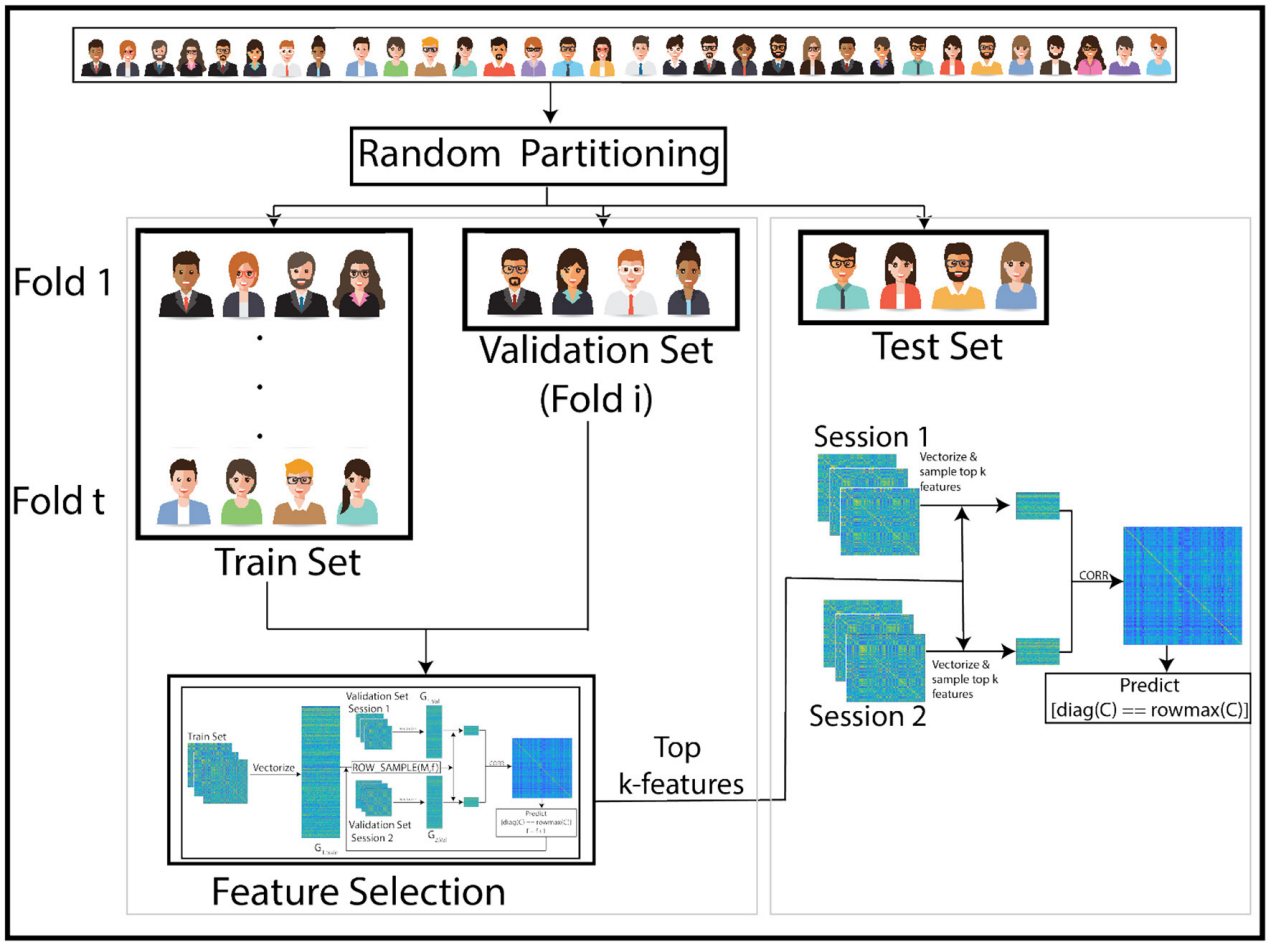
Overview of our pipeline: The subjects are randomly partitioned into train and test sets in each of 1,000 repetitions. Training is performed with 10-fold cross-validation within the train set. High leverage score features are selected using the train set and training performance is assessed using a validation subset (1-fold of the train set). In each iteration, a different fold within the train set is held back for validation. Optimal features identified using this training procedure are then used to match images across sessions of subjects in the test set. The pipeline for training process is in Figure 2.

**Figure 2:**
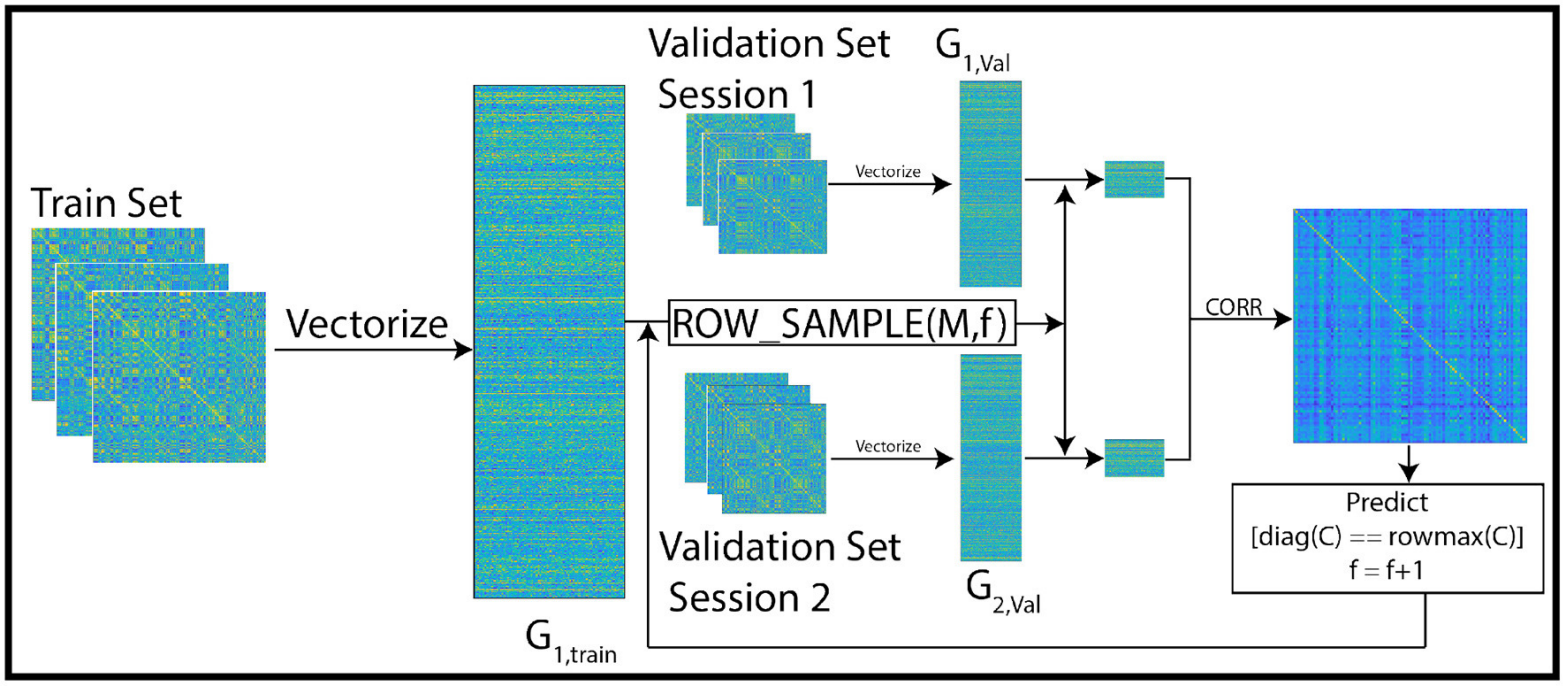
Overview of the training pipeline: the first session of the train set is vectorized and stacked into the matrix *G*_1, *train*_. In each iteration, *f* features are selected from the vectorized train set using leverage score sampling. Then, the feature space of the vectorized validation set (a subset of the train set) matrices is restricted to the selected subset of features. The correlation between pairs of columns of the sub-sampled validation matrix is used to predict identity across sessions. In each iteration, the size of the feature set is incremented upto a maximum of 100. The optimal feature set is the one with maximum prediction accuracy. This feature set is then used in our experiments to predict identity of subjects in the test set.

To ensure robustness, we repeat this experiment for 1,000 different (random) subsets of subjects. In each repetition, we randomly shuffle the subjects into train and test sets. The training procedure is performed with 10-fold cross validation as shown in Figures 1, 2. We cycle through the folds, holding back a different subset of the train set as validation set in each iteration. The features are selected on the train-subset and prediction accuracy is calculated on the validation set. To show that the features generalize well to the remaining population, we predict identity on the hold-out test set, using the restricted feature space. Table 1 presents accuracy for different sizes of train/test splits. The results are averaged over all folds and over 1,000 repetitions. We note from the table that the accuracies obtained in the reduced space are comparable to those obtained using the full correlation matrices, or by using a low rank matrix approximation—indicating that our small set of features captures essentially all of the distinguishability in the data. For the rest of our experiments, we use an 80/20 split, but repeat the cross-validation procedure to ensure statistical robustness of our results. The overall pipeline and the training procedure are shown in Figures 1, 2.

**Table 1:** Accuracy of principal features subspace method for different splits of training and test samples.

**Train/test split**	**Train accuracy (%, mean ± std)**	**Test accuracy (%, mean ± std)**
80/20	96.23 ± 2.24	93.11 ± 3.61
50/50	96.30 ± 2.59	92.94 ± 3.82
30/70	96.81 ± 3.07	90.23 ± 4.30
20/80	97.01 ± 3.22	87.60 ± 5.27
10/90	97.72 ± 2.65	81.86 ± 7.15

*The results show consistently high train accuracy across a range of training set sizes. As expected, training accuracy increases with smaller training sets; however, test accuracy goes down as training set size is reduced, due to overfitting*.

To characterize the statistical significance of the selected features, we repeat the experiment with the same number of features chosen uniformly at random 10^6^ times. The average accuracy was much lower (~55%, see Table 2). None of these instances yielded accuracy values higher that for our leverage-score based feature selection. This suggests high statistical significance of our identified features (empirical *p*-value < 10^−6^).

**Table 2:** Performance of leverage-score based feature selection technique, compared to selecting features uniformly at random.

**Feature selection method**	**Training accuracy (%, mean ± std)**	**Test accuracy (%, mean ± std)**
Leverage score	96.23 ± 2.24	93.11 ± 3.61
Random	54.62 ± 7.76	54.52 ± 7.52

*The significantly higher test and training set accuracy demonstrates the performance of our principal subspace feature selection method*.

### 3.2. The Feature Set Is Robust Across Individuals

Our first set of experiments identified a set of features from individuals in *G*_1_, and used these features to draw correspondence (identification) between the two groups. Note that in these experiments, the identification of signatures and identification of subjects is on the same set of individuals. In our second set of experiments, we show that this feature-set is invariant across individuals. Specifically, we identify features using one set of subjects (features with highest leverage scores in the training set), and use these features to draw correspondences between a distinct set of subjects (the test set) across the two groups. The result of this experiment is presented in Table 2. The high test-set accuracy indicates that features relevant for the training set are also discriminative for the test set (*p*-value: < 10^−6^). This suggests an anatomical and/or physiological basis for the features selected.

We further highlight the implication of this result: it is possible to apply a computational procedure to extract features from training and test sets. The computational procedure may be a complex function defined over all features. This is in fact the case with prior methods. In contrast, what our experiment shows is that no such computational procedure is required—rather, a robust selection of sample-invariant features is highly discriminative and accurate.

### 3.3. The Size of the Discriminating Feature Set Is Compact

**Figure 3:**
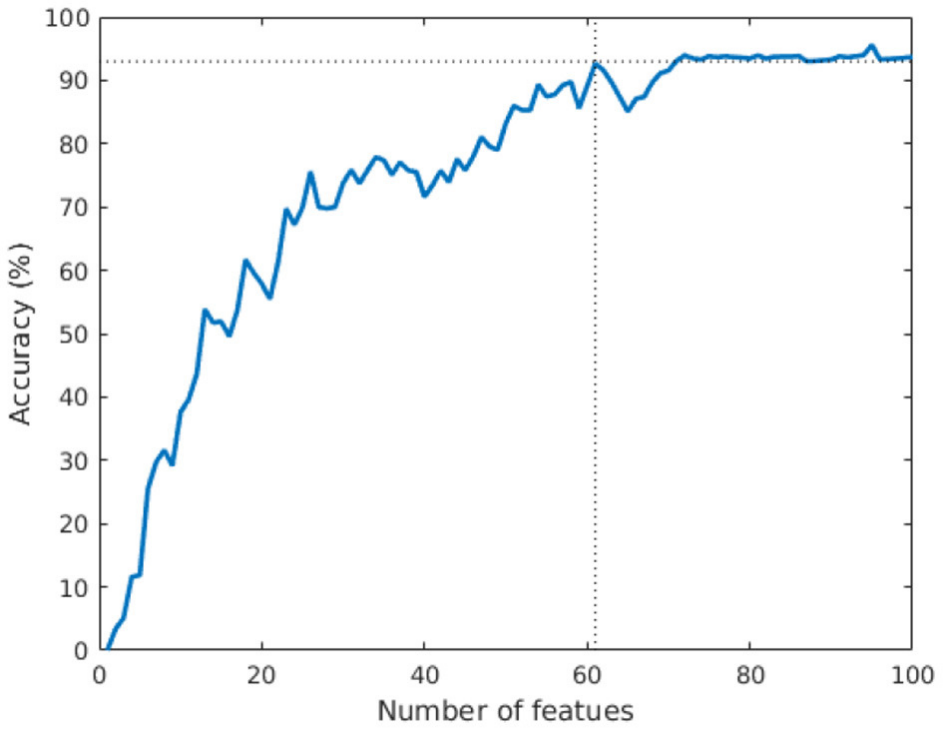
Variation of test accuracy as a function of feature set size. The rapid convergence in accuracy demonstrates that a small set of features codes the discriminating signature.

Our first set of experiments demonstrated that a small set of 100 features is robust and statistically significant in its discriminative power. We now seek to determine the smallest such set that can code individual signatures with high accuracy. We design an experiment in which the number of features is successively increased (in order of decreasing leverage scores) and the test accuracy is measured at each step. The result of this experiment is presented in Figure 3. We note that the accuracy essentially plateaus using 61 features at 93%. The maximum accuracy (95.5%) is achieved using 95 features. Finally, we also note that restricting the feature-space to the single top leverage-score feature is not expressive in terms of prediction accuracy. This is expected, as single features are not expressive by themselves. Rather, a small subset of features is responsible for encoding the signature—a fact that we explore further in our next result.

It is important to note that this experiment relies on a leverage score ordering of the features. It is indeed possible that an alternate ordering may yield a smaller feature set. However, our results on the suitability of leverage scores for feature selection, combined with the rapid plateauing of accuracy, strongly suggest that a feature set comprised of 61–95 features (from among over 64K features) is enough to code individual signatures with high accuracy.

### 3.4. A Small Number of Structural Regions Encode the Signature

Our previous experiments used entries in the correlation matrix (edges in correlation network among regions of the brain) as features. We now investigate whether a small number of structural features (regions in the brain) is capable of coding individual signatures with high discriminating power.

**Figure 4:**
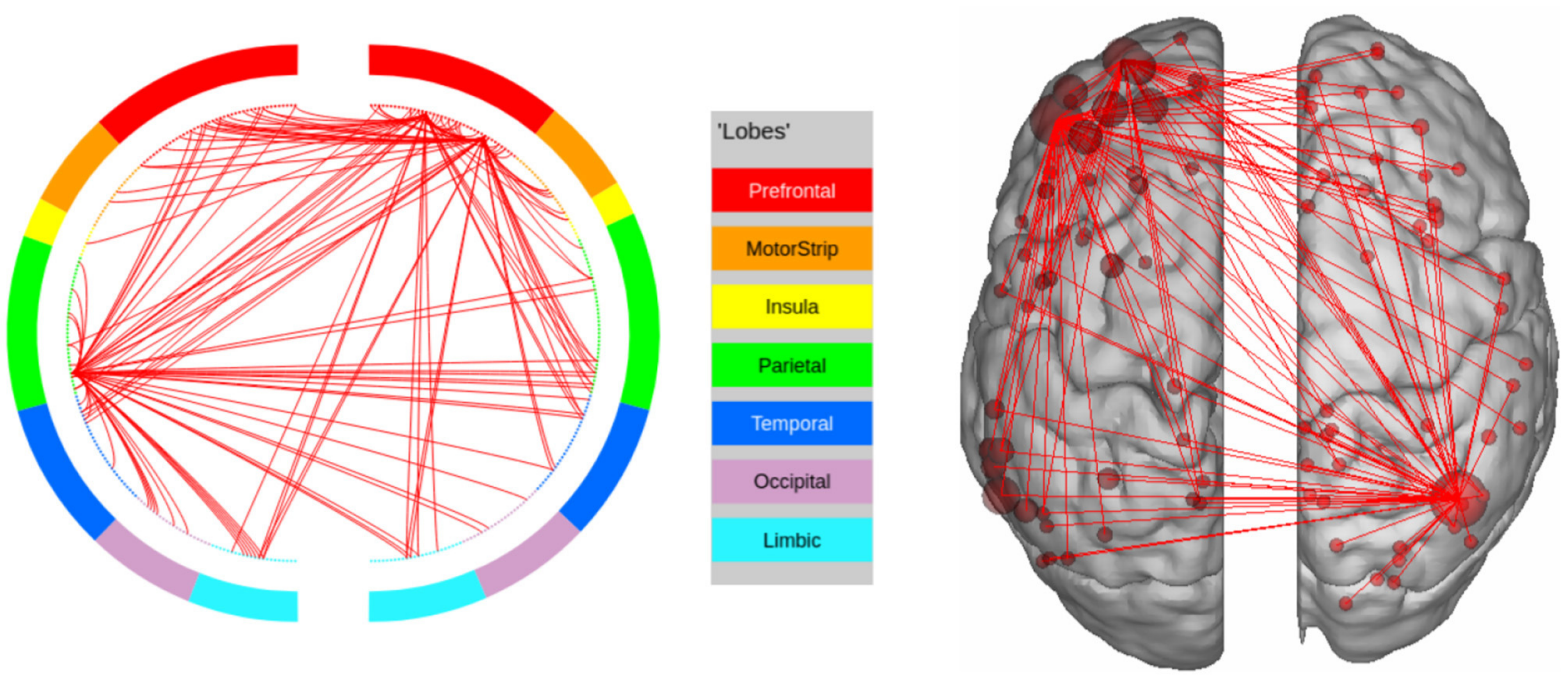
High confidence edges that encode resting-state signature in the human connectome. The connectivity map shows that the signature is strongly expressed in the prefrontal cortex and the parietal cortex. For illustrative purposes, we show edges when at least one terminal node has a degree of 30 (These visualizations were created using BioImage Suite https://www.nitrc.org/projects/bioimagesuite/).

In this experiment, we compute the top 100 features with the highest leverage scores for a randomly selected subset of 80 subjects. We repeat this procedure 1,000 times. We then extract the high confidence features using a hyper-geometric *p*-value cutoff of 10^−20^ which gives a set of 302 features. The results are shown in Figure 4. We observe that the prefrontal cortex and the parietal regions strongly encode the signature. This is in agreement with Finn et al. (2015), who showed that the fronto-parietal network has the most discriminating power. However, in contrast to previous methods, we are able to make this observation solely by analyzing the resting state connectomes of one session in the training set, as opposed to other methods (such as the on due to Finn et al., 2015) which require connectomes from both sessions to identify the implicated regions.

In order to find the regions based on our parcellation scheme, we select the regions that are over-represented in the top features across all 1,000 tests (hypergeometric *p*-value < 10^−20^). This process resulted in 12 high-confidence regions, which, as we show, encode the signature. To do this, we restricted ourselves to work with the 24 × 24 connectomes, corresponding to both hemispheres of the previously identified regions. We then constructed the vectorized representation of this smaller matrix for the test subjects and found the accuracy to be 94.05 (±1.22). In comparison, identifiability accuracy for 12 randomly chosen regions was 41.47 (±12.25, for *p*-value < 10^−6^). The regions in this set are listed in Table 3. Overall, the signature expressing regions occupy about 4.5% of the cortex.

**Table 3:** Top brain regions associated with discriminating features for resting-state connectomes.

**Area number**	**Area notation**	**Region**
141	Temporo-Parieto-Occipital Junction 3	Parietal
11	Premotor Eye Field	Premotor
95	Intraparietal dorsal	Parietal
85	Area Anterior 9-46v	Prefrontal
86	Area 9-46v	Prefrontal
97	Inferior 6-8 transitional area	Prefrontal
146	Intra Parietal 0	Parietal
48	Lateral Intra Parietal Ventral	Parietal
89	Area Anterior 10p	Prefrontal
87	Area 9 anterior	Prefrontal
137	PHT	Lateral temporal
79	IFJa	Inferior frontal

*We observe that most of the signature expressing regions are in the prefrontal and the parietal cortex*.

Structurally, five of the 12 regions (namely Area Anterior 9-46v, Area 9-46v, Inferior 6-8 transitional area, Area Anterior 10p, and Area 9 anterior) belong to the Dorsolateral Prefrontal Cortex. Furthermore, Intraparietal dorsal, Intraparietal 0, and Lateral Intra Parietal Ventral belong to the Parietal Cortex (see Supplementary Material in Glasser et al., 2016). This shows that regions coding the signature are physiologically localized.

### 3.5. Our Method Outperforms State of the Art Methods in Terms of Test Accuracy

In this set of experiments, we compare the accuracy of our method with other state-of-the-art techniques. Finn et al. (2015) compute all pairwise Pearson correlation coefficients, where each data point is represented by all elements of the upper diagonal of the time-series correlation matrix. We randomly choose 80 out of 100 subjects and compute pairwise similarity; the accuracy of Finn et al.'s method is 88.65 (±1.76) averaged over 1,000 trials. Amico and Goñi (2018) first denoise the data by retaining a subset of the principal components. As before, we randomly choose 80 subjects and compute the training set accuracy (see Table 4). We observe that removing the top few principal components improves accuracy, since these components correspond to signals that are common to all subjects, as noted in Amico and Goñi (2018). The test set accuracy is computed by removing the top principal components of the training dataset from the test dataset. This reveals that the common signal of the training dataset is an artifact of those particular subjects, and not a pattern that provides significant insights about the functional behavior of the brain. In all cases, our method yields comparable training set accuracy, significantly higher test set accuracy, while, importantly, relying on a small set of structurally and functionally interpretable features.

**Table 4:** Comparison of training and test accuracy for various methods.

**Principal components**	**Training accuracy (%, mean ± std)**	**Test accuracy (%, mean ± std)**
All [same as Finn et al. (2015)]	88.65 ± 1.76	88.98 ± 1.72
2:end	94.30 ± 1.35	71.76 ± 8.76
11:end	96.74 ± 1.00	69.61 ± 8.94
21:end	95.03 ± 1.90	69.44 ± 8.99
31:end	71.97 ± 6.08	68.95 ± 9.07
41:end	72.77 ± 1.74	65.70 ± 9.59
Principal features subspace	96.23 ± 2.24	93.11 ± 3.61

*Numbers in the first row use the entire matrix; the next five rows use a subset of the principal components; the last row corresponds to our method. Our method achieves almost optimal training set accuracy and significantly better test set accuracy compared to competing methods*.

### 3.6. Individuals Performing Tasks Can Be Distinguished

**Figure 5:**
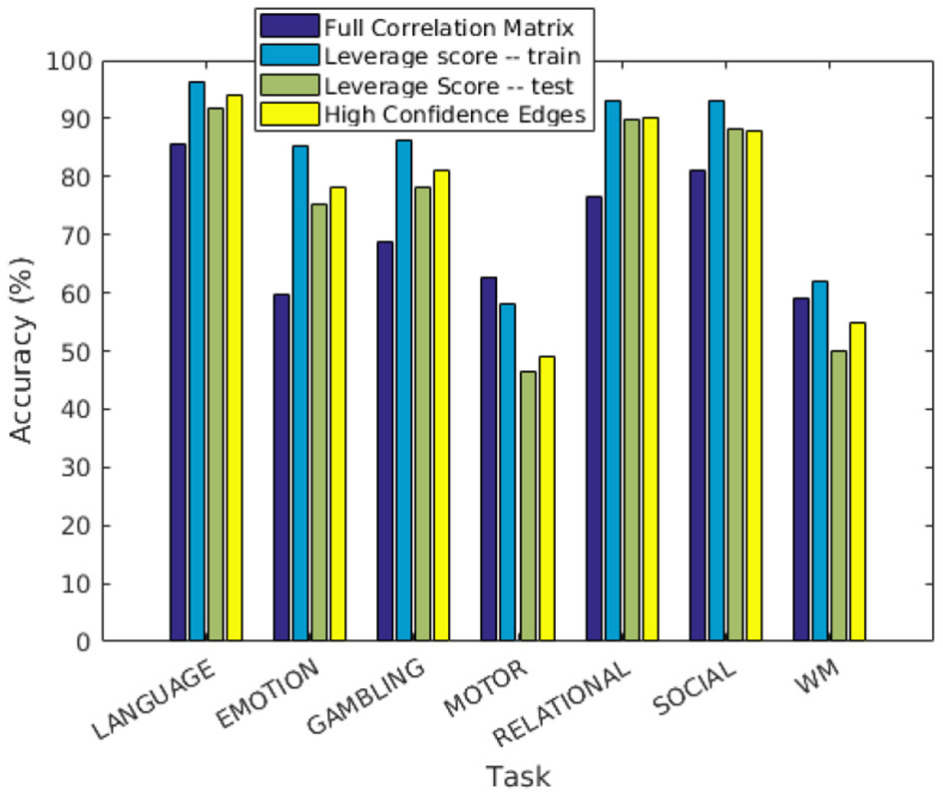
Prediction accuracy of individuals performing tasks.

In this experiment, we demonstrate that our method for finding discriminating edges extends to the case of task-based functional networks. For each task, we construct the two group matrices and partition subjects into training and test sets as described before. We compute leverage scores separately for each task using the respective training subsets, restrict the feature space to top leverage scores, and report predictions on the test set in the same manner as in the resting state experiments. The results (averaged over 1,000 trials) are shown in Figure 5. The performance of our approach is comparable to using the full correlation matrix in all but two tasks, namely MOTOR and WM. This indicates that our method recognizes features that are important for most tasks. As before, we localize the features to physical regions on the brain. In order to do this, we only use features whose leverage scores are consistently sufficiently high to remain in the reduced feature space across all 1,000 trials (hyper-geometric *p*-value < 10^−20^).

**Figure 6:**
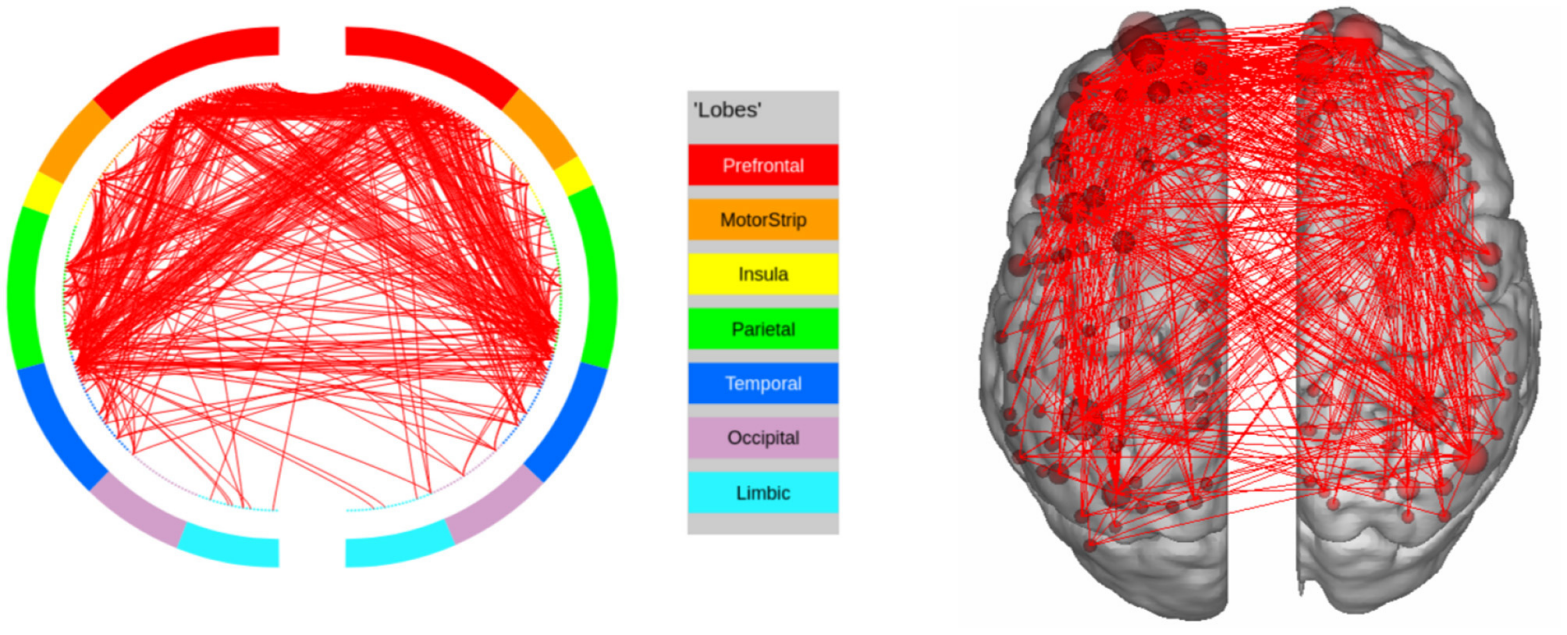
High confidence edges that encode signature while performing the language task of HCP. The regions with high edge density are in good agreement with the regions of interest (ROIs) for language (Barch et al., 2013). For illustrative purposes, we show edges when at least one terminal node has a degree of 30.

We further discuss the results for each task. In the LANGUAGE task, the statistically significant regions identified by our method include the Inferior Frontal Cortex and surrounding regions (Orbital and Frontal Cortex and Dorsolateral Prefrontal Cortex). They also include regions in the Anterior Cingulate and Medial Prefrontal Cortex, as well as areas that surround the Superior Temporal Cortex (the Medial and Lateral Temporal Cortex). These results are consistent with the regions of interest for LANGUAGE, identified by Barch et al. (2013). Furthermore, the task involves auditory inputs, and requires communication via pressing a button. This explains the Premotor Cortex and the Auditory Association Cortex being recognized as regions of interest by our approach. The high confidence edges for the task are shown in Figure 6.

**Figure 7:**
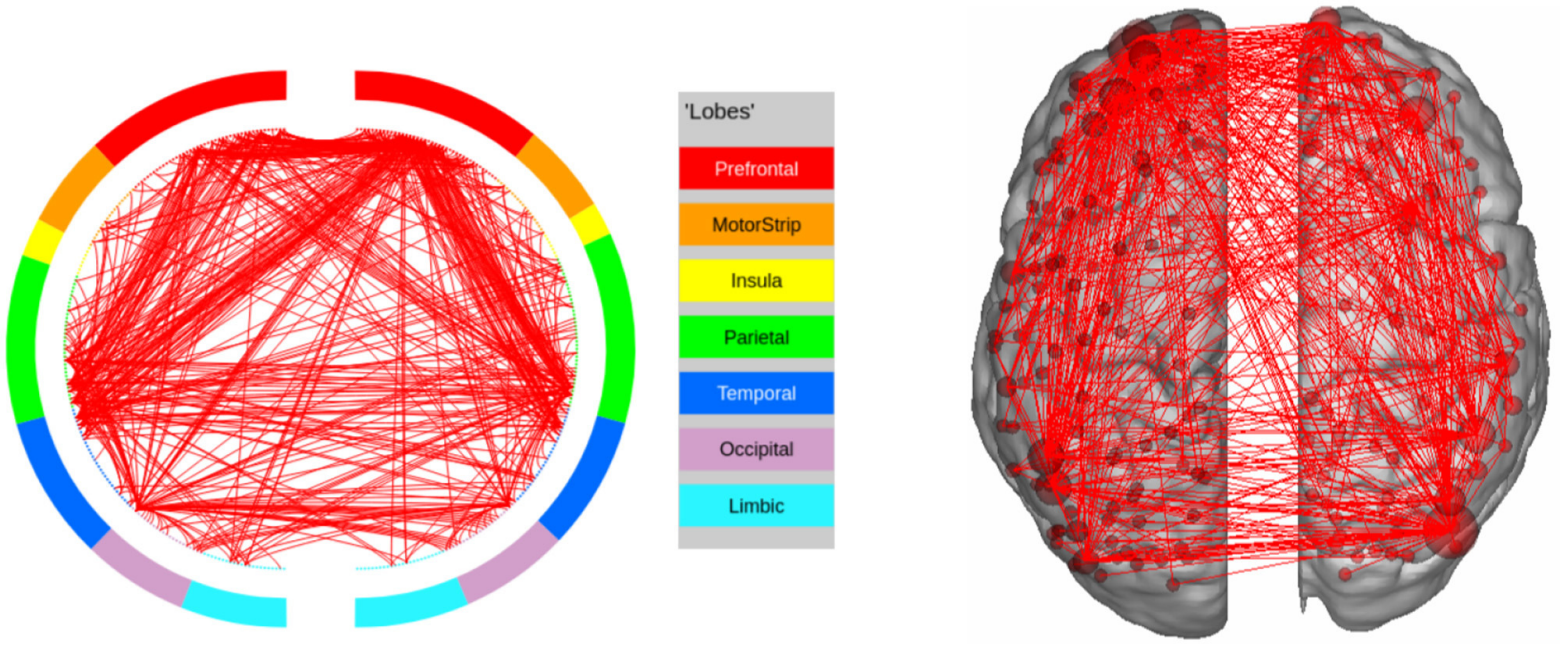
High confidence edges that encode signature while performing the emotion processing task of HCP. The regions with high edge density are in good agreement with the ROIs for emotion processing (Barch et al., 2013). For illustrative purposes, we show edges when at least one terminal node has a degree of 30.

In EMOTION, the regions implicated in the signature are found in the Prefrontal Cortex and neighboring regions (Inferior Frontal Cortex, Orbital and Polar Frontal Cortex), as well as areas surrounding the Insula (Lateral and Temporal Parietal Cortex). These results are consistent with the results of Barch et al. (2013), except for the fact that we do not identify the Medial Temporal Cortex as a region of interest. The connectivity graph for EMOTION is shown in Figure 7.

**Figure 8:**
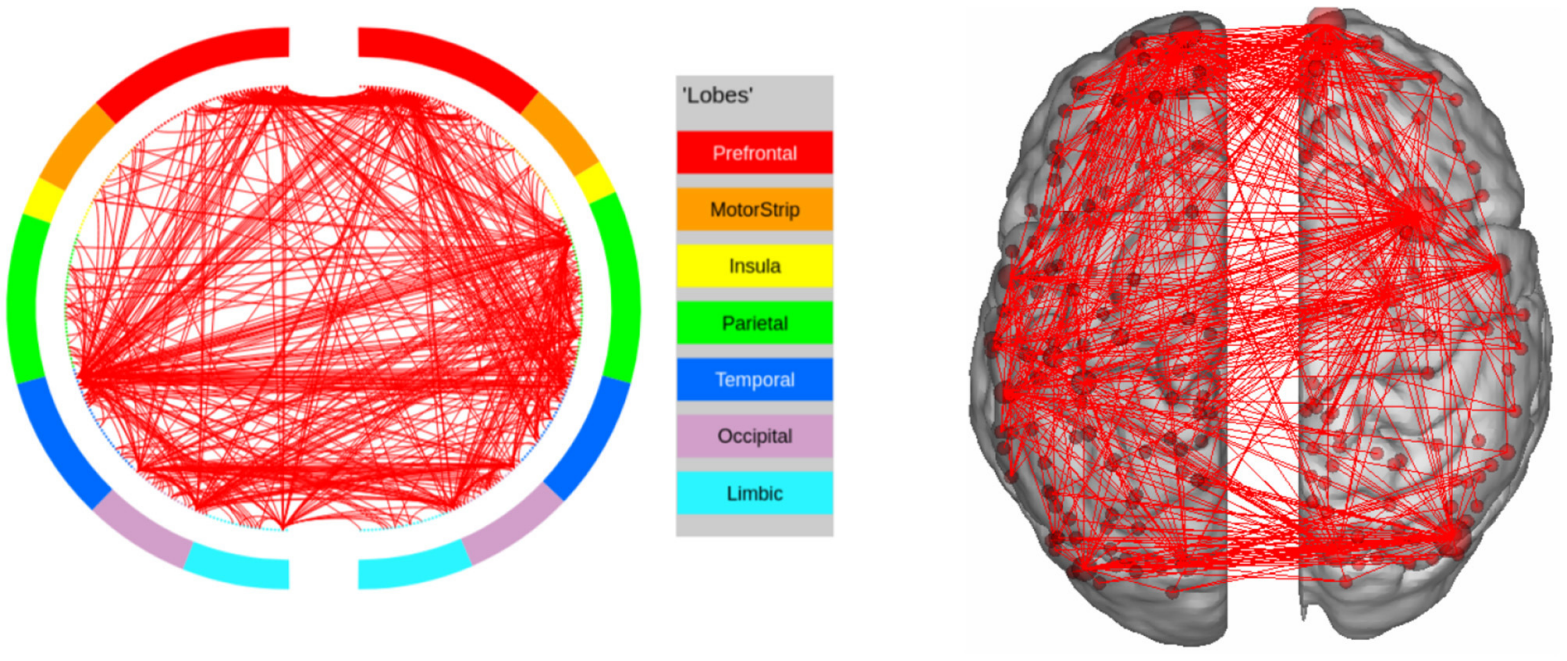
High confidence edges that encode signature while performing the gambling task of HCP. The regions with high edge density are in good agreement with the ROIs for gambling (Barch et al., 2013). For illustrative purposes, we show edges when at least one terminal node has a degree of 30.

In GAMBLING, we find the expected regions of interest in the Prefrontal cortex, as well as the Orbito-frontal cortex. The other area of interest is the Sub-cortical Striatum, which is not included in our parcellation scheme. In this experiment, the subjects are shown visual stimuli and are required to communicate their decision by pressing a button (Figure 8). This explains the fact that the regions in Visual and Motor Cortex are being recognized as potential regions of interest.

**Figure 9:**
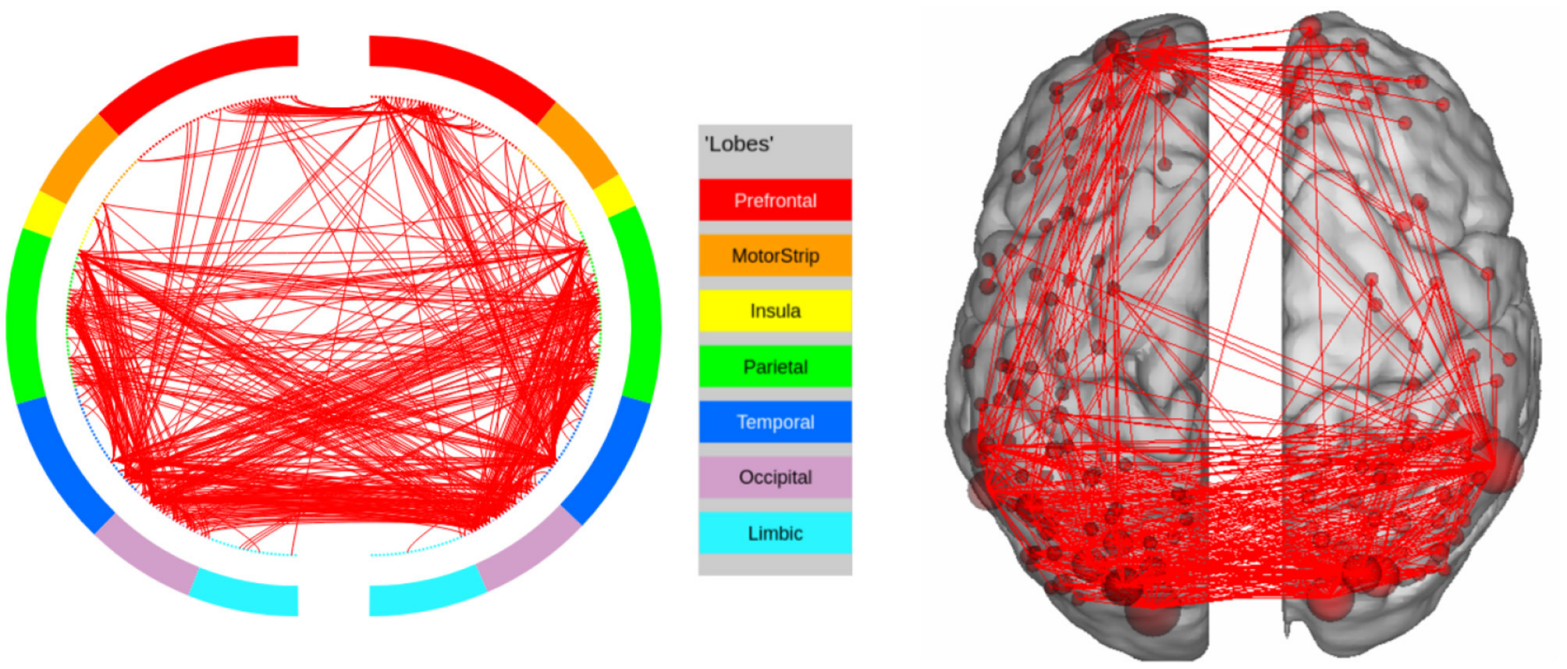
High confidence edges that encode signature while performing the social processing task of HCP. The regions with high edge density are in good agreement with the ROIs for social processing. For illustrative purposes, we show edges when at least one terminal node has a degree of 30.

**Figure 10:**
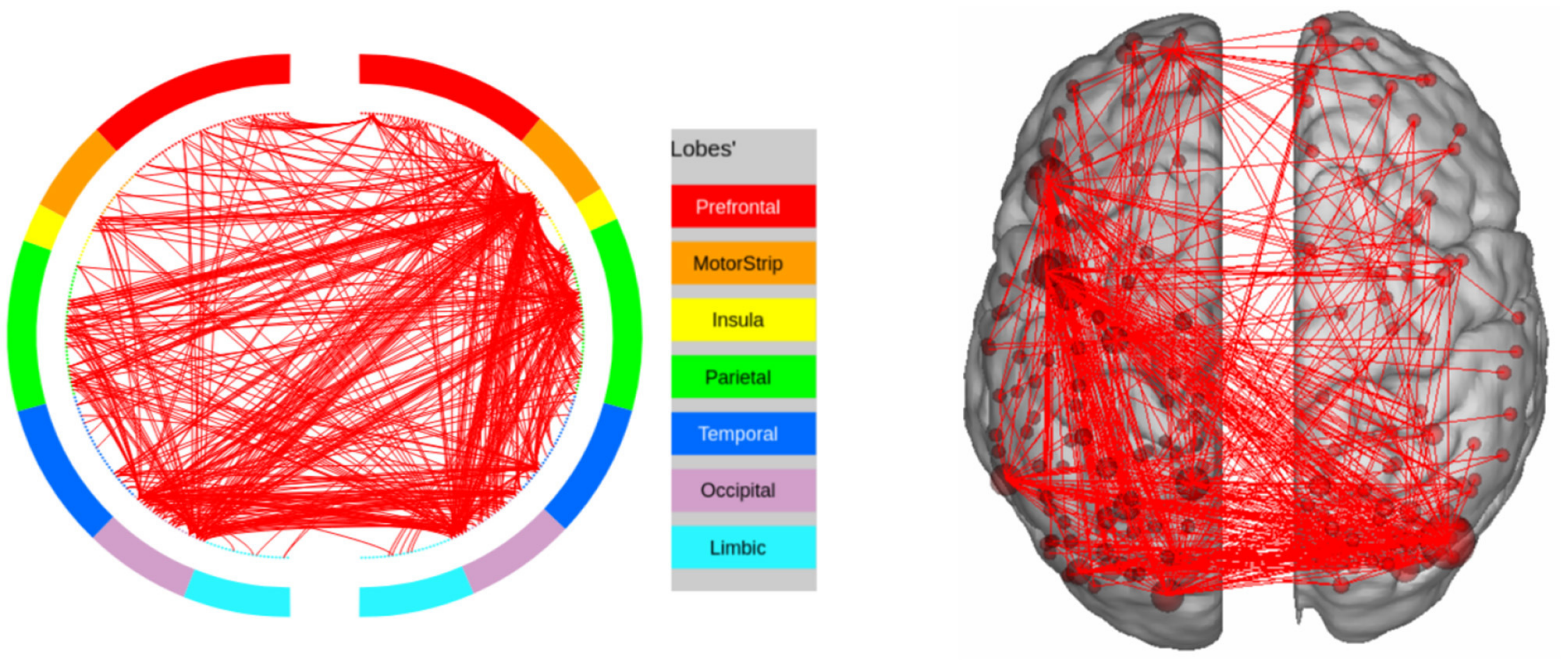
High confidence edges that encode signature while performing the relational processing task of HCP. The regions with high edge density are in good agreement with the ROIs for relational processing. For illustrative purposes, we show edges when at least one terminal node has a degree of 30.

**Figure 11:**
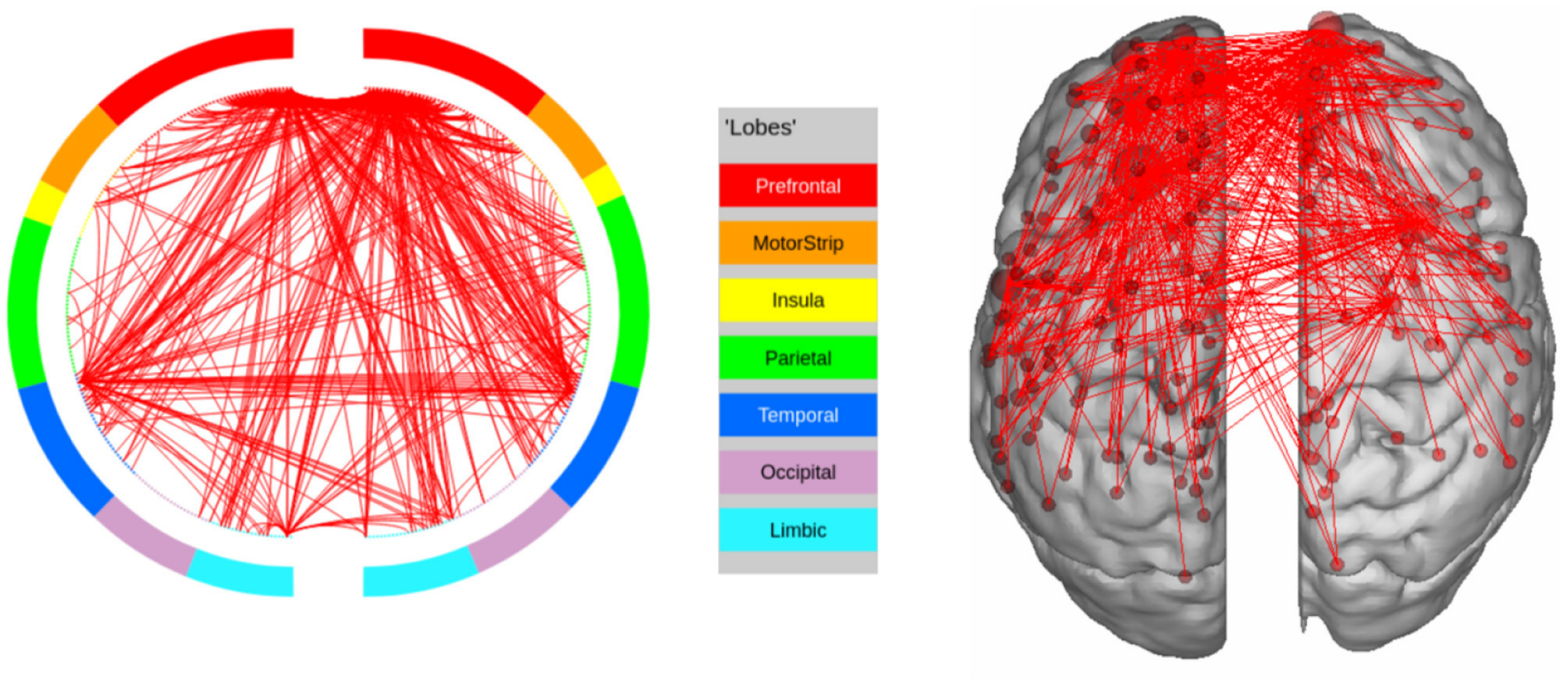
High confidence edges that encode signature while performing the language task of HCP. Our method does not find the regions implicated by Barch et al. (2013) in this case. For illustrative purposes, we show edges when at least one terminal node has a degree of 30.

**Figure 12:**
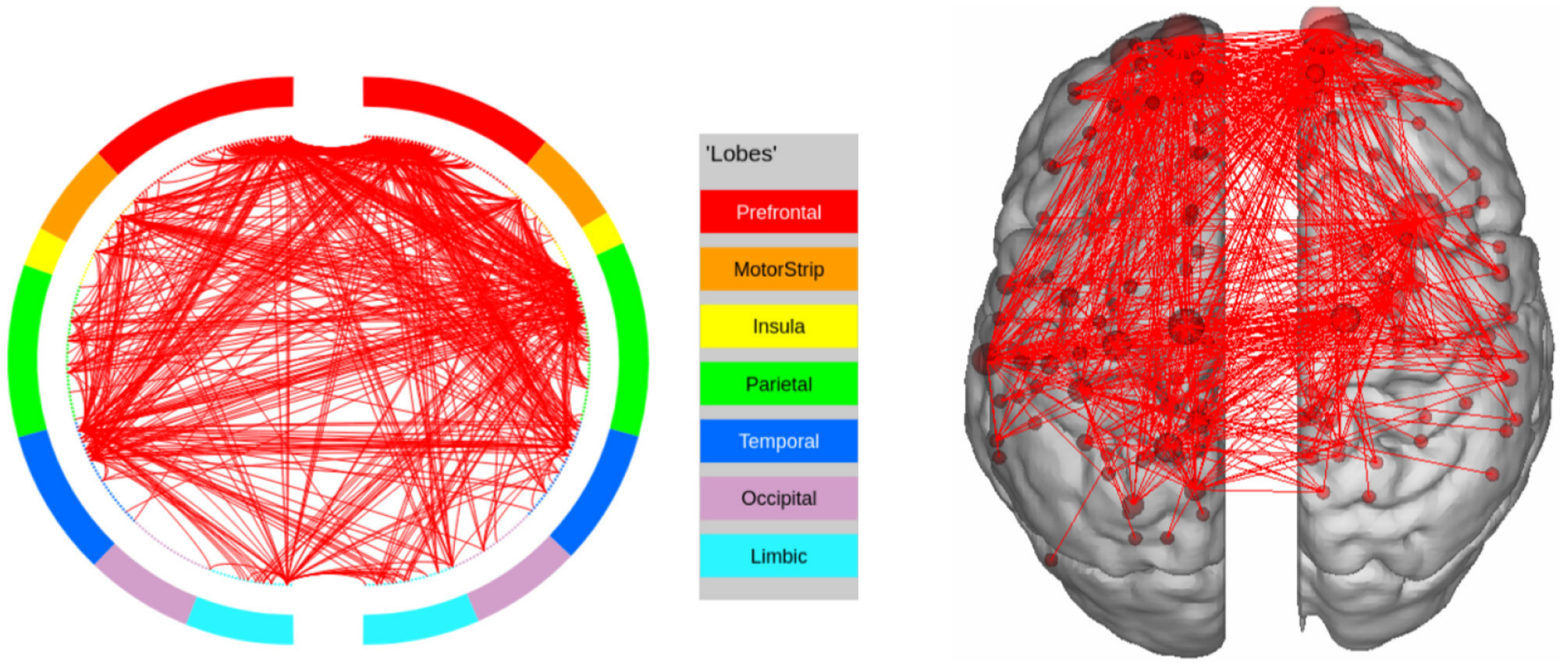
High confidence edges that encode signature while performing the working memory task of HCP. Our method does not find the regions implicated by Barch et al. (2013) in this case. For illustrative purposes, we show edges when at least one terminal node has a degree of 30.

The results for SOCIAL tasks are shown in Figure 9: we identify all relevant regions of interest (Temporal Parietal Junction and Medial Prefrontal Cortex, along with the visual cortex and the motor cortex). In the RELATIONAL task, we identify regions in the prefrontal cortex, but also in the parietal cortex, as shown in Figure 10. In the MOTOR task (Figure 11), we do not identify some regions of interest (Somatosensory and Motor cortex). This explains the relatively poor prediction accuracy for this task, and WM (Figure 12).

While the regions obtained by our method broadly agree with the regions that are expected to be activated during respective tasks, we note that a general pipeline of tasks for fMRI would factor in additional information, such as presence/absence of various stimuli, duration of each block, and performance metrics associated with the tasks performed. The connectivity profiles of each of the tasks are sufficiently distinct, suggesting that we can identify tasks, which is indeed our main conclusion.

### 3.7. Tasks Are Identifiable With High Accuracy

In this final experiment, we identify the tasks performed by an individual. For each subject, we create two group matrices. The first group matrix contains the LR encoding of all tasks and the second group matrix contains the corresponding RL encoding. Here, a match is successful if the same tasks across the two groups are more similar to each other than with any other task. As before, we split the subjects randomly into train and test sets of size 80 and 20, respectively. For each subject in the train set, we find the features corresponding to the top leverage scores. This gives us 80 sets of features. We find the set of statistically significant features for the entire training set using a hyper-geometric *p*-value test (threshold 10^−50^) to identify recurring features across all subjects. These statistically significant features are used to restrict the feature space in the test set. The accuracy, in terms of correctly identified tasks in the test set, averaged across 1,000 trials was 91.93 (±1.34%). In contrast, the same experiment using the entire connectome yielded accuracy of 84.95 (±2.2%).

## 4. Discussion

In this paper, we present a novel matrix sampling method to answer critical questions related to individual and task-specific signatures in the human brain connectome. We show that a small set of features in a functional connectome codes for the identity of an individual. Furthermore, we show that these features (edges in the connectome) are robust, statistically significant, and invariant across individuals. The regions corresponding to these features are consistent with existing literature—both for resting-state and task, supporting the physiological basis for our method.

The core contribution of our method lies in the fact that it can predict relevant features, without requiring multiple scans. In doing so, it generalizes the application of brain signatures beyond the HCP dataset. Specifically, it can be used in studies where the goal is to find individual differences in a cohort. Furthermore, since the method is built on a sound theoretical foundation, its computational and statistical underpinnings are well-characterized.

The success of leverage scores in picking relevant features for brain signatures suggests that it can also be used in conventional fMRI studies, where the aim is to find differences between cases and controls. The key distinction between the two setups is that the former requires unsupervised feature selection, whereas the latter requires supervised feature selection. We note that this necessitates new sampling techniques, along with associated theoretical guarantees.

We also identify a few limitations of our study, which provide avenues for future research: (i) our work only uses the HCP dataset. Future studies may use other datasets and different acquisition protocols to further establish the robustness of our methods; (ii) it is unclear as to what is the optimal spectral range to consider (i.e., which columns of *U* must we use to compute Leverage scores). This choice can have an impact on the selected features. While our current work uses a default selection, there is potential for further improvement by refining the selection procedure; (iii) the stability of signatures is related to the session time. In this context, it is useful to determine the minimum session time required to find stable signatures; and (iv) certain tasks (MOTOR and WM) are associated with poor prediction accuracy. Further study is required to ascertain the cause of this loss of accuracy, and methods to improve it.

## Data Availability Statement

Publicly available datasets were analyzed in this study. This data can be found at: www.humanconnectomeproject.org.

## Ethics Statement

Ethical review and approval was not required for the study on human participants in accordance with the local legislation and institutional requirements. The patients/participants provided their written informed consent to participate in this study.

## Author Contributions

VR, PD, and AG conceptualized the problem. VR setup the experiments with inputs from PD and AG. VR and AG wrote the papers with inputs from PD. All authors contributed to the article and approved the submitted version.

## Conflict of Interest

The authors declare that the research was conducted in the absence of any commercial or financial relationships that could be construed as a potential conflict of interest.

## Footnotes

^1^https://github.com/Washington-University/HCPpipelines.
